# Digital engagement and adolescent wellbeing: insights from the UK millennium cohort study

**DOI:** 10.1093/eurpub/ckag128

**Published:** 2026-08-06

**Authors:** Miaoqing Yang, Charles Opondo, Yang Wang, Adam Martin

**Affiliations:** Academic Unit of Health Economics, Leeds Institute of Health Sciences, Faculty of Medicine and Health, University of Leeds, Leeds, United Kingdom; Department of Medical Statistics, Faculty of Epidemiology and Population Health, London School of Hygiene & Tropical Medicine, London, United Kingdom; Faculty of Health and Wellness, City University of Macau, Macau, China; China Center for Health Development Studies, Peking University, Beijing, China; Academic Unit of Health Economics, Leeds Institute of Health Sciences, Faculty of Medicine and Health, University of Leeds, Leeds, United Kingdom

## Abstract

Existing research on screen time and adolescent psychological wellbeing often overlooks how digital engagement interacts with other daily activities. Uncertainty remains about which activities are complemented or displaced by digital use, and whether specific combinations of activities are beneficial or detrimental to wellbeing. We used detailed time-use diary data from 3,288 participants in the UK Millennium Cohort Study, collected during the age-14 survey. Adolescents recorded their activities in 144 consecutive 10-min slots over a 24‑h period, selecting from 44 pre-specified activity types. Latent profile analysis was used to identify distinct time-use patterns. This approach accounts for the interdependence and trade-offs among different activities, rather than examining each of them in isolation. Associations between these profiles and later psychological wellbeing, measured using the Strengths and Difficulties Questionnaire, were examined using multinomial logistic regression. Three time-use profiles emerged: (i) ‘Frequent digital users’ (21.0%), with high digital engagement, especially gaming, more common among males and those from lower socioeconomic backgrounds, showing poorer psychological wellbeing; (ii) ‘Physically active, moderate digital users’ (59.7%), who balanced digital and non-digital activities with high physical activity, showing more favourable psychological wellbeing; and (iii) ‘In-person socialisers, low digital users’ (19.3%), with low digital use and high face-to-face interaction, showing intermediate level of psychological wellbeing. Findings suggest that a balanced mix of daily activities is associated with more favourable adolescent wellbeing, highlighting the importance of promoting overall activity balance rather than focusing on digital time alone.

## Introduction 

Adolescent mental health has become a growing public health concern, with approximately one in five children and young people aged 8–25 in the UK identified as having a probable mental disorder in 2023 [1]. This growing crisis has prompted increased research into its underlying causes, with one area of particular focus being the role of children’s time allocation across different types of activities [2–4]. In recent years, as digital devices increasingly shape how adolescents communicate, access information, and spend their leisure time, concerns have emerged about their potential negative effects on psychological wellbeing [5]. Excessive digital time may displace essential activities such as sleep, physical exercise, and in-person social interactions, all of which are crucial for mental health [6]. It may also expose young people to online harassment, violent content, and unrealistic social comparisons, which can contribute to anxiety, depression, and reduced life satisfaction [7, 8]. The potential impact of digital engagement on adolescent wellbeing has been the subject of ongoing and highly scrutinized debates in academia, media, and political sphere, with policymakers in several countries, including the UK, considering measures such as banning smartphones in schools and restricting social media access for those under 16 [9–11]. Leading health institutions (e.g. the UK Royal College of Paediatrics and Child Health) have issued guidelines on recommended screen time levels to mitigate potential risks associated with excessive digital engagement [12–14].

Despite widespread concerns, existing research provides mixed evidence on the association between digital time and adolescent psychological wellbeing [15, 16]. While some studies report a significant negative relationship [8, 17–19], others find little to no clear evidence of harm [20, 21]. The association varies depending on the type of digital activity, population subgroups, amount of time spent, and whether engagement is active or passive. Additionally, findings differ by research data sources and methods used to examine these relationships. Most studies rely on self-reported time-use data, as well as cross-sectional and correlational designs, which are susceptible to recall bias and reverse causality [22]. In addition, previous research has largely focused on the amount of time spent on a specific digital activity of interest, framing the debate around whether increased or decreased digital time alone directly influences adolescent wellbeing. However, focusing on digital time in isolation neglects the broader context of time use within a fixed 24-h time constraint, failing to account for the interdependence and trade-offs between competing daily activities.

The fixed constraint is a key concept in economic theories of resource allocation, which suggest individuals distribute their budget of time (and money) to maximize personal utility [23]. While financial constraints are less relevant for adolescents, time remains a limited resource: every hour spent on one activity is an hour not spent on another. Additionally, digital activities can compete with or complement other forms of daily engagement. For instance, an adolescent who spends hours gaming may engage less in physical activity, whereas another who connects with active peers on social media may be encouraged to spend more time outdoors. To better understand the impact of digital engagement, more comprehensive approaches are needed to examine how digital engagement fits into the broader context of daily time use.

In this study, we used 24-h time-use diary data from the UK Millennium Cohort Study (MCS) to examine how adolescents allocate their time across digital engagement and a range of other activities. Rather than treating digital time in isolation, we applied latent profile analysis (LPA) to identify distinct time-use patterns and examine their associations with psychological wellbeing. By identifying latent subgroups based on individuals’ time allocation patterns, LPA captures population heterogeneity and reveals how activities interact and are traded off within a 24-h time budget, rather than examining each behaviour as an independent exposure, as in traditional regression approaches. Although LPA has been used to examine activity patterns and wellbeing among adults and older populations, particularly in relation to remote work and caregiving [24–26], it has not been applied to adolescent wellbeing. Existing studies using LPA on adolescents’ activity patterns often rely on accelerometer data and focus primarily on obesity-related outcomes [27, 28].

This study addresses three research questions (RQs):

1. What are the distinct patterns of time-use allocation among adolescents?
2. How are these distinct time-use patterns associated with demographic and socioeconomic characteristics?
3. Are distinct time-use patterns associated with variations in psychological wellbeing?

## Methods

### Data description

The MCS, one of Britain’s most renowned national longitudinal birth cohort studies, tracks the lives of approximately 19,000 children born in the UK between 2000 and 2001. To date, participants have been surveyed at six waves: at nine months and at ages 3, 5, 7, 11, 14, and 17 years. The study covers a wide range of topics, including demographics, socioeconomic status, and children’s physical, mental health and developmental outcomes.

Time use survey data were collected during the age-14 survey of the MCS and recorded the timing and duration of all daily activities that occurred throughout two specified full days: one weekday and one weekend day. The survey split the day into 144 10-min time slots starting from 4 a.m. each day. There were 44 pre-specified activity items to be chosen by the respondents, providing granular insights into a broad range of activities.

The sample for the current study consists of 3,288 cohort members who completed the weekend time-use diary. We focused on weekend activities, as adolescents have limited discretionary time during weekdays due to structured school schedules. This is evident in our data, where weekday time-use patterns showed minimal variation, with the majority of time allocated to school-related activities. In addition, adolescents who reported more leisure activities on weekdays were likely surveyed during school holidays or were not attending school at the time, meaning that weekday activity patterns are not directly comparable across participants and may introduce bias. In contrast, weekends provide considerably more flexibility and autonomy, allowing adolescents to allocate their time across a wider range of activities. As such, weekend data provide a more accurate reflection of adolescents’ preferences towards leisure activities, making it more appropriate for examining time-use patterns in relation to psychological wellbeing.

Attrition and item non-response are recognized sources of potential bias in longitudinal surveys such as the MCS. We used multiple imputation by chained equations (MICE) to generate 20 imputed datasets [29]. The imputation model included all variables used in the main analyses. Regression analyses were conducted separately within each imputed dataset, and estimates were combined using Rubin’s rules to obtain pooled coefficients and standard errors.

### Variables

The 44 pre-specified activity items are categorized in the MCS time use diary user guide into eleven general activities (i.e. sleep and personal care, education-related activities, work, house chores, eating and drinking, physical exercise and sports, traveling, social events/outings, digital media, spiritual/volunteering activities, hobbies/relaxation, and a category for missing time inputs) [30]. We further consolidated these categories into 6 main groups for use in the LPA (i.e. sleep, physical activities, education-related activities, social and hobbies, digital time, personal care, travel and household chores), following the classification adopted by Atkin et al. [31]. Supplementary Table S1 provides details on the domains used in this study and the activity items included within each domain.

Psychological wellbeing outcomes were measured using the Strengths and Difficulties Questionnaire (SDQ), a validated tool for identifying socioemotional and behavioural problems among children and adolescents across various contexts, including school, home, and clinical settings [32, 33]. It consists of 25 items grouped into five subscales: (i) conduct problems, (ii) emotional problems, (iii) hyperactivity/inattention, (iv) peer relationship problems, and (v) prosocial behaviour. Each subscale is scored from 0 to 10 based on responses to five questions. The Total Difficulties Score (TDS) is calculated by summing the first four subscales, as they reflect psychological challenges—higher scores indicate greater difficulties. In contrast, the prosocial behaviour subscale captures positive traits such as empathy, kindness, and helpfulness. Therefore, it was analysed separately from the TDS to provide insight into an adolescent’s social competence and emotional maturity. We used SDQ data reported by adolescents at ages 14 and 17, with age 14 scores included as baseline measures and age 17 scores as outcomes (summary statistics are provided in Supplementary Table S2). Adjusting for baseline SDQ allows us to account for prior behavioural and emotional characteristics, thereby capturing earlier influences of potential confounding factors.

Covariates were selected based on prior research on adolescent activity and psychological wellbeing [31, 34]. The variables included age, sex, ethnicity (white vs. non-white), obesity status (based on body mass index; binary), high-income households (based on equivalized family income quintiles: highest two quintiles vs. others), two-parent household (binary), parental employment status (both parents employed vs. others), parental education (National Vocational Qualification level 3 or above vs. others), home ownership, and the Index of Multiple Deprivation (IMD). Summary statistics of covariates are shown in Supplementary Table S3.

### Data analyses

For RQ1, we applied LPA to empirically identify distinct time-use patterns across various activity types and create subgroups of adolescents with similar activity profiles. The time spent on each activity type was measured by the number of 10-min time slots allocated to that activity. Latent profiles were identified using robust maximum likelihood estimation, iterating across two to five latent profiles. We limited the number of profiles to a maximum of five to allow for the identification of interpretable and meaningful patterns without overfitting the data. As the number of profiles increases, model fit typically improves; however, too many profiles may capture idiosyncratic features of the sample rather than underlying population structure, thereby reducing generalizability and replicability [35]. The optimal number of profiles was determined using the Akaike Information Criterion (AIC), Bayesian Information Criterion (BIC), Sample Size Adjusted Bayesian Information Criterion (ssaBIC) and entropy index. Lower values of AIC, BIC, and ssaBIC indicate better model fit, while higher entropy denotes better profile separation [36]. We also considered the interpretability of the resulting profiles and the proportion distribution of the sample as models with extremely small profiles were considered inappropriate [37, 38]. Once the latent profiles that best fit the data were identified, individuals were assigned to the most probable time-use pattern based on their highest membership probability. This probabilistic profile assignment, while not explicitly accounting for classification uncertainty, has been widely adopted in LPA [35, 39].

We also examined how different types of digital activities (including sending emails, texting and messaging, using social media, video calling, using video or audio content, or playing games) contributed to each identified profile. The analyses provided insights into the role of specific type of digital technologies in shaping overall time-use behaviours.

For RQ2, we examined how the identified activity profiles varied across key demographic, socioeconomic and health characteristics, which allowed us to explore potential inequalities in time-use patterns. To assess the predictive power of these covariates on profile membership, we conducted a multinomial logistic regression, regressing profile membership on all covariates. Marginal effects of the covariates were subsequently calculated at each level of predicted probability of profile membership.

For RQ3, we investigated the relationship between time-use patterns at age 14 and SDQ outcomes at age 17 to see how different activity profiles are associated with adolescents’ psychological wellbeing over time. The analysis was conducted in three stages: an unadjusted model, a partially adjusted model controlling for SDQ outcomes at age 14, and a fully adjusted model that accounted for SDQ outcomes at age 14, demographic, socioeconomic and health characteristics.

All analyses were conducted using Stata/MP version 18.0 (codes are available upon request).

## Results

### Descriptive statistics

Table 1 summarizes the percentage of participants engaged in each activity domain (participation rate) and the average time spent daily on these activities (mean and standard deviation) among those who participated. Activities are ranked based on total time spent, calculated by multiplying the number of participants by the average time spent per day. Apart from sleep, which had the highest participation and duration, digital engagement was the activity to which adolescents devoted the second most time, with 91.1% of adolescents engaging in it for an average of 299 min per day. Supplementary Table S1 shows a detailed breakdown of activity items within this domain: watching TV or videos (69.1%) and playing electronic games (36.3%) had the highest participation rates and the longest durations—averaging 178 and 190 min respectively among participants. Social media use (40.8%) and general internet browsing (15.7%) were also frequent, though with lower average durations. In contrast, engagement in physical activity was notably lower, with participation rates ranging from 5% to 17% depending on the activity type.

**Table 1.** Participation rate and time spent daily for each activity domain on weekend days (*N* = 3288)

Activity domains	Participation rate *n* (%)^a^	Time spent (mean and SD)^b^
Sleep	3273 (99.54%)	623 (134)
Digital time	2994 (91.06%)	299 (184)
Personal care, travel and household chores	3247 (98.75%)	227 (143)
Social and hobbies	2799 (85.13%)	252 (172)
Physical activities	1835 (55.81%)	131 (122)
Education-related activities	990 (30.11%)	118 (116)

^a^Participation rate: cohort members spending >0 min on each activity.

^b^Daily minutes spent on each activity by those participating.

### RQ1: Time use allocation patterns

LPAs were conducted using six activity domains and the corresponding fit statistics are presented in Supplementary Table S4. Although the four-profile model has the lowest AIC, BIC and ssaBIC values, it included one very small latent profile comprising only 3% of the sample, which fell well below the recommended threshold of 10% to avoid over-extraction [38]. Therefore, the three-profile model was identified as the best fit for the data and used for further analysis. The entropy value of this model was 0.79, approaching the 0.80 threshold recommended in the literature, indicating adequate classification certainty of the profiles [36].

**Figure 1.**
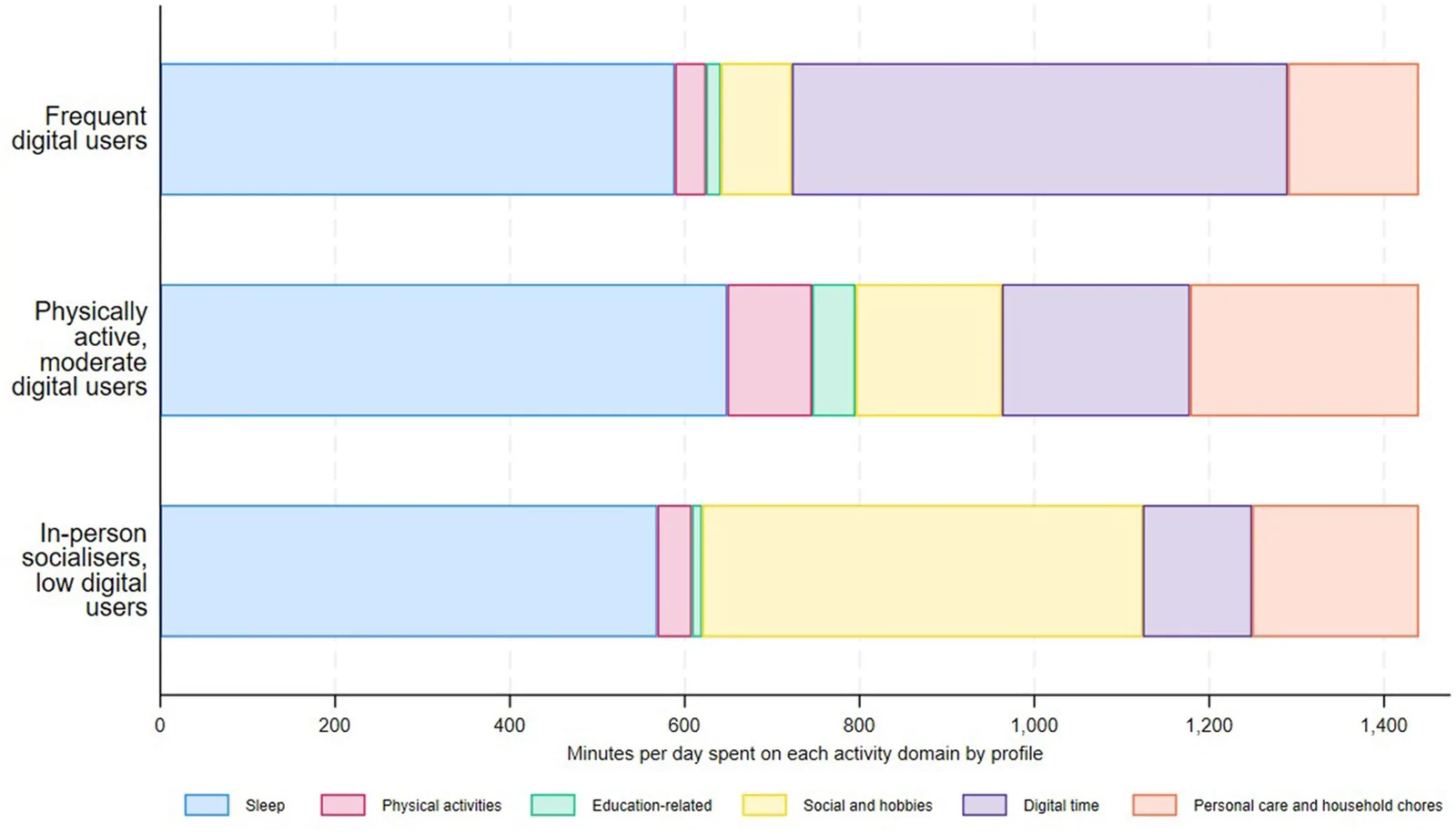
Minutes per day spent on each activity domain by latent profiles.

The findings from the three-profile model are shown in Fig. 1 (more details summarized in Supplementary Table S5). Three distinct profiles of adolescents’ time-use patterns related to digital use were identified:

1. Profile 1—‘Frequent digital users’ (*N* = 691, 21.0%): This group spent the most time on digital activities (an average of 567 min a day) while engaging the least in offline social activities (e.g. face-to-face socializing, shopping, cinema, religious activities), as well as hobbies (including reading) and relaxation. They also had the lowest levels of physical activity and below-average sleep duration. A detailed view of the specific digital activity young people engaged in is shown in Supplementary Fig. S1. The high level of digital involvement was mainly driven by time spent playing electronic games and watching TV.
2. Profile 2—‘Physically active, moderate digital users’ (*N* = 1962, 59.7%): Representing the majority of participants, this group maintained a relatively well-balanced lifestyle, dividing their time approximately evenly between digital devices and social activities, hobbies, and relaxation. They had lower levels of digital engagement (215 min) alongside higher levels of physical activity and more time spent on personal care, travel, and household tasks. They exhibited the highest levels of engagement in education-related activities, physical exercise, and sleep, reflecting an overall active and structured routine.
3. Profile 3—‘In-person socialisers, low digital users’ (*N* = 635, 19.3%): This group prioritized offline social interactions, hobbies, and relaxation, dedicating the most time to face-to-face social activities. They spent the least time on digital media (125 min) and sleep across the three profiles, and also reported relatively low levels of physical activity.

### RQ2: Associations with demographic and socioeconomic characteristics

Table 2 presents the marginal effects from the multinomial logistic regression for the predictors of latent profile membership (summary statistics are provided in Supplementary Table S6). ‘Frequent digital users’ were 15% (95% CI: −0.18 to −0.12; *P* < .001) less likely to be female and 7% (95% CI: −0.10 to −0.03; *P* = .001) less likely to have both parents in employment. They also tended to live in more deprived areas compared to the other groups (*β* = −0.01; 95% CI: −0.01 to −0.00; *P* = .002). In contrast, ‘Physically active, moderate digital users’ and ‘In-person socialisers, low digital users’ had a higher proportion of females and were slightly more advantaged socioeconomically.

**Table 2.** Marginal effects from the multinomial logistic regression of latent profile membership (*N* = 3148)

	‘Frequent digital users’ (*N* = 691)	‘Physically active, moderate digital users’ (*N* = 1962)	‘In-person socialisers, low digital users’ (*N* = 635)
Age	0.01	0.01	−0.02
95% confidence interval	[−0.02, 0.04]	[−0.03, 0.05]	[−0.05, 0.02]
* p* value	*0.632*	*0.701*	*0.328*
Proportion of females	−0.15	0.06	0.09
95% confidence interval	[−0.18, −0.12]	[0.03, 0.10]	[0.06, 0.12]
* p* value	*0.000*	*0.000*	*0.000*
Proportion of white ethnicity	0.05	−0.08	0.03
95% confidence interval	[0.01, 0.09]	[−0.13, −0.03]	[−0.01, 0.07]
* p* value	*0.023*	*0.003*	*0.170*
Proportion of obesity	0.01	−0.01	−0.00
95% confidence interval	[−0.05, 0.07]	[−0.08, 0.06]	[−0.06, 0.05]
* p* value	*0.671*	*0.805*	*0.908*
Proportion of two-parent households	0.00	−0.01	0.01
95% confidence interval	[−0.04, 0.05]	[−0.07, 0.05]	[−0.04, 0.06]
* p* value	*0.912*	*0.705*	*0.722*
Proportion of both parents employed	−0.07	0.04	0.02
95% confidence interval	[−0.10, −0.03]	[−0.01, 0.09]	[−0.02, 0.07]
* p* value	*0.001*	*0.109*	*0.229*
Proportion of high household income	0.02	−0.00	−0.02
95% confidence interval	[−0.02, 0.06]	[−0.05, 0.05]	[−0.06, 0.02]
* p* value	*0.291*	*0.965*	*0.297*
Proportion of high parental education	−0.02	0.02	0.00
95% confidence interval	[−0.06, 0.01]	[−0.03, 0.06]	[−0.03, 0.04]
* p* value	*0.222*	*0.409*	*0.852*
Proportion of house ownership	−0.02	0.04	−0.02
95% confidence interval	[−0.06, 0.02]	[−0.01, 0.09]	[−0.06, 0.02]
* p* value	*0.401*	*0.126*	*0.284*
IMD deciles	−0.01	0.01	0.00
95% confidence interval	[−0.01, −0.00]	[0.00, 0.01]	[−0.00, 0.01]
* p* value	*0.002*	*0.038*	*0.605*

Results shown as marginal effects *β* and 95% confidence intervals in square brackets, along with *P* values.

IMD: Index of multiple deprivation.

### RQ3: Associations with psychological wellbeing

Table 3 presents the associations between latent profile membership and SDQ scores at age 17 (summary statistics are provided in Supplementary Table S6). ‘Frequent digital users’ consistently showed evidence of higher TDS across all models compared to ‘physically active, moderate digital users’ (the reference group). In the fully adjusted model, the TDS was 0.59 points higher (95% CI: 0.21–0.97; *P *= .002), indicating a modest but statistically meaningful increase in psychological difficulties. ‘In-person socialisers, low digital users’ also demonstrated higher TDS (*β* = 0.62, 95% CI: 0.24–1.01; *P *= .002). Across the five domains, ‘frequent digital users’ were strongly associated with greater difficulties, particularly in emotional (*β* = 0.23, 95% CI: 0.06–0.40; *P *= .007), hyperactivity (*β* = 0.21, 95% CI: 0.06–0.37; *P *= .005) and peer problem (*β* = 0.19, 95% CI: 0.05–0.33; *P *= .007) domains, even after full adjustment. They also showed lower prosocial behaviour, although this association became non-significant in the fully adjusted model. ‘In-person socialisers, low digital users’ also exhibited higher difficulties in emotional domain (*β* = 0.28, 95% CI: 0.11–0.45; *P *= .001), but associations with hyperactivity and peer problems were weaker and did not reach conventional significance levels in the fully adjusted model. Estimates for prosocial behaviour were also negative but not statistically significant. Results from the multiple imputation analyses were broadly consistent with the complete-case analyses (Supplementary Table S7).

**Table 3.** Associations between latent profiles and SDQ scores

	Unadjusted	**Partially adjusted** ^a^	**Fully adjusted** ^b^
SDQ total difficulties score			
Frequent digital users	1.24	0.53	0.59
95% confidence interval	[0.74, 1.73]	[0.17, 0.90]	[0.21, 0.97]
* p* value	*0.000*	*0.004*	*0.002*
In-person socializers	0.73	0.67	0.62
95% confidence interval	[0.22, 1.24]	[0.29, 1.05]	[0.24, 1.01]
* p* value	*0.005*	*0.001*	*0.002*
SDQ conduct score			
Frequent digital users	0.15	0.06	0.04
95% confidence interval	[0.03, 0.27]	[−0.05, 0.16]	[−0.07, 0.14]
* p* value	*0.018*	*0.273*	*0.500*
In-person socializers	0.11	0.08	0.08
95% confidence interval	[−0.01, 0.24]	[−0.03, 0.18]	[−0.02, 0.19]
* p* value	*0.084*	*0.150*	*0.130*
SDQ emotional score			
Frequent digital users	0.31	0.19	0.23
95% confidence interval	[0.11, 0.51]	[0.02, 0.35]	[0.06, 0.40]
* p* value	*0.002*	*0.027*	*0.007*
In-person socializers	0.34	0.33	0.28
95% confidence interval	[0.14, 0.55]	[0.16, 0.50]	[0.11, 0.45]
* p* value	*0.001*	*0.000*	*0.001*
SDQ hyperactivity score			
Frequent digital users	0.36	0.21	0.21
95% confidence interval	[0.17, 0.55]	[0.06, 0.36]	[0.06, 0.37]
* p* value	*0.000*	*0.005*	*0.005*
In-person socializers	0.19	0.15	0.14
95% confidence interval	[−0.00, 0.39]	[−0.01, 0.30]	[−0.01, 0.29]
* p* value	*0.056*	*0.059*	*0.076*
SDQ peer score			
Frequent digital users	0.42	0.20	0.19
95% confidence interval	[0.26, 0.58]	[0.07, 0.33]	[0.05, 0.33]
* p* value	*0.000*	*0.003*	*0.007*
In-person socializers	0.09	0.13	0.12
95% confidence interval	[−0.08, 0.25]	[−0.01, 0.27]	[−0.02, 0.26]
* p* value	*0.304*	*0.068*	*0.094*
SDQ prosocial score			
Frequent digital users	−0.34	−0.17	−0.10
95% confidence interval	[−0.50, −0.18]	[−0.30, −0.04]	[−0.24, 0.03]
* p* value	*0.000*	*0.012*	*0.134*
In-person socializers	−0.13	−0.09	−0.08
95% confidence interval	[−0.29, 0.03]	[−0.23, 0.04]	[−0.22, 0.06]
* p* value	*0.122*	*0.180*	*0.266*
Number of observations	**2712**	**2667**	**2570**

^a^Adjusted for baseline SDQ scores.

^b^Adjusted for baseline SDQ scores, demographic, socioeconomic and health characteristics.

The reference group is the ‘physically active, moderate digital users’ profile.

Results are shown as marginal effects *β* and 95% confidence intervals in square brackets and p values in italics.

## Discussion

### Key findings

This study aimed to identify distinct time-use patterns among adolescents, examine their associations with demographic and socioeconomic factors, and assess whether these patterns are associated with differences in prospective psychological wellbeing. A three-profile model provided the best fit to the data: (i) ‘Frequent digital users’ (21.0%) spent the most time on digital activities while engaging the least in social activities, hobbies, and physical exercise. (ii) ‘Physically active, moderate digital users’ (59.7%), the largest group, demonstrated a balanced time-use pattern, dividing their time relatively evenly between digital and non-digital leisure activities. They exhibited the highest levels of engagement in education-related activities, physical exercise, and sleep. (iii) ‘In-person socialisers, low digital users’ (19.3%) prioritized offline social interactions, hobbies, and relaxation while spending significantly less time on digital media and physical activities. They also had the shortest sleep duration among the three profiles. ‘Frequent digital users’ were more likely to be male and come from disadvantaged background. This profile was associated with relatively poorer psychological outcomes, exhibiting higher levels of emotional, hyperactivity, and peer difficulties, higher TDS, and lower prosocial behaviour, even after adjusting for SDQ scores at age 14 and a wide range of covariates. ‘In-person socialisers, low digital users’ also demonstrated elevated difficulties, particularly in emotional problem domain, but with less consistent significance.

### Comparisons with previous findings

The magnitude of the associations observed in our study should be interpreted in the context of prior research. Atkin et al. (2021) reported relatively small differences in TDS, around 0.1–0.2 points, associated with reallocating time between daily activities (e.g. adding 15 min of physical activity or reducing electronic media use) [31]. These smaller estimates likely reflect their focus on marginal time reallocations across the whole sample, whereas our study compared subgroups with distinct time-use patterns, which may capture more pronounced differences in daily routines and their links to mental health. Khan et al. (2023) reported TDS reductions of approximately 0.5–1.8 points associated with sports participation [34]. By comparison, the increases in TDS observed in our fully adjusted models—0.59 for ‘frequent digital users’ and 0.62 for ‘in-person socialisers, low digital users’—fall within the same general range, albeit on the positive (worsening) side of the scale. Evidence from other analyses focusing on well-established socioeconomic determinants, such as family income, are associated with slightly smaller differences (about 0.23–0.48) in TDS compared to our estimates after adjustment for confounding factors [40]. Therefore, the magnitude of the associations observed in our study is non-negligible, indicating that patterns of digital engagement may play a meaningful role in adolescent psychological wellbeing.

### Strengths and limitations

This study has several key strengths. First, it exploits detailed 24-h time-use diary data from a large, nationally representative cohort of UK adolescents. Although self-reported, these data are generally considered more accurate than retrospective survey measures, as they capture activities contemporaneously and reduce recall bias. Second, the use of LPA enables distinct time-use patterns to be identified, capturing the heterogeneity in adolescents’ daily routines rather than treating the population as homogeneous. This approach also crucially enables examination of how digital engagement fits within the 24-h time constraint and interacts with other activities, such as physical exercise, sleep and offline social interactions, rather than focusing solely on digital time. Third, previous literature on adolescent digital engagement and psychological wellbeing has primarily relied on cross-sectional designs [22, 31]. A key strength of this study is to examine how time-use patterns at age 14 are associated with psychological wellbeing at age 17, which helps mitigate concerns about reverse causality. In addition, we used multiple imputation to address attrition across waves, providing a robustness check for the stability of our findings. Finally, by accounting for a broad range of demographic and socioeconomic factors, this study provides insights into how time-use behaviours may be shaped by structural inequalities, offering valuable evidence to inform targeted interventions and policies.

Several limitations are noted. First, time-use diary data were collected at a single time point in the MCS about a decade ago. Although types of digital activities among adolescents may have evolved since then, the broader implications regarding how these activities interact with other daily activities are likely to remain applicable to contemporary contexts, as the importance of balancing digital, physical, and social activities extends beyond specific platforms or technologies. Second, our analysis was restricted to weekend time-use data, which does not capture the full range of adolescents’ daily activities. However, weekday time-use patterns may be difficult to interpret, as variations in leisure activities are likely driven by whether data were collected during school holidays or term-time, which may introduce bias and reduce comparability. Third, the measurement of digital engagement is limited to time spent using digital media and does not capture other aspects such as content or platform-specific features. Future research would benefit from the use of repeated time-use diary data and technology-enabled data (e.g. smartphone-based tracking) to provide a more accurate and detailed picture of adolescents’ activities.

### Implications

Our findings suggest that having a well-balanced mix of activities—including physical exercise, adequate sleep, and digital and non-digital social interactions—is associated with better adolescent psychological wellbeing than simply reducing digital time. While ‘frequent digital users’ showed the least favourable mental health outcomes, ‘physically active, moderate digital users’, who balanced digital engagement with diverse activities, showed more favourable wellbeing. Interestingly, ‘in-person socialisers, low digital users’, despite low levels of digital engagement, did not consistently exhibit the most favourable outcomes, indicating that reduced screen use alone does not necessarily correspond to better psychological wellbeing. The finding challenges the narrow focus of most existing research, which examined digital time in isolation and assumed that less is always better. Instead, our findings highlight that moderate digital use, when integrated with other positive activities, is not inherently harmful. The broader time-use pattern and balance of activities are more relevant to adolescent wellbeing. Policy approaches solely aimed at restricting digital time may be overly simplistic, overlooking the need to promote a balanced mix of daily activities.

This study contributes to the ongoing debate on digital engagement by emphasizing the heterogeneity in adolescents’ time-use patterns and their differing impacts on wellbeing. The findings underscore the importance of considering digital use within the broader context of daily activities rather than viewing it as an isolated factor. While ‘frequent digital users’ experienced poorer emotional and behavioural outcomes, those with more balanced engagement across social, educational, and physical activities demonstrated better psychological wellbeing. Interventions could focus on promoting a balanced lifestyle, encouraging physical and social engagement alongside moderate digital use. Additionally, addressing socioeconomic disparities—particularly for disadvantaged groups—may help reduce excessive reliance on digital devices by expanding access to alternative recreational opportunities.

## Conclusions

This study highlights the diverse ways adolescents allocate their time and how these patterns are associated with psychological wellbeing. By identifying different time-use patterns, our findings emphasize the need for tailored interventions that promote activity diversity while accounting for socioeconomic factors. These insights provide a foundation for evidence-based policies that integrate health, education, and social strategies to foster healthier time-use patterns and support adolescent mental health.

## Supplementary Material

ckag128_Supplementary_Data

## Data Availability

The data analysed in this study are from the UK Millennium Cohort Study. These data are publicly available via the UK Data Service to registered users, subject to the relevant data access agreements. Key points This study moves beyond screen time alone to examine how digital engagement interacts with other daily behaviours within a 24-h framework. Frequent digital use is linked to increased psychological difficulties. Lower socioeconomic status is linked with more digital use, particularly gaming. Promoting healthy combinations of behaviours, such as physical activity, sleep, and social interaction, may be more effective than simply limiting screen time. Insights are timely given rising concerns about adolescent mental health and ongoing policy debates around digital use.
