# Supplementary material for: Digital engagement and adolescent wellbeing: insights from the UK millennium cohort study
Source: Eur J Public Health. 2026 Aug 6;36(5):ckag128. doi: 10.1093/eurpub/ckag128 (PMC13445974; doi:10.1093/eurpub/ckag128)
Supplement: Supplementary file 1 [file ckag128_supplementary_data.zip › ejph-2025-09-om-0825-File007.docx]

**Table S1: Types and amount of weekend activities (N=3,288)**

| **Activity domains** | **Activity items** | **Participation rate (cohort members spending >0 minutes on each activity)**  **n (%)^a^** | **Daily minutes spent on each activity by those participating (mean and standard deviation)^b^** |
| --- | --- | --- | --- |
| Digital media | Watching TV, DVDs, downloaded videos | 2,271 (69.07%) | 178 (122) |
|  | Playing electronic games and Apps | 1,193 (36.28%) | 190 (168) |
|  | Browsing and updating social networking sites (e.g. Twitter, Facebook, BBM, Snapchat) | 1,341 (40.78%) | 84 (94) |
|  | General internet browsing, programming (not time on social networking sites) | 515 (15.66%) | 96 (109) |
|  | Listening to music, radio, iPod, other audio content | 624 (18.98%) | 73 (72) |
|  | Answering emails, instant messaging, texting | 528 (16.06%) | 67 (88) |
|  | Speaking on the phone (including Skype, video calls) | 266 (8.09%) | 75 (84) |
| Sleep | Sleeping and resting (including sick in bed) | 3,273 (99.54%) | 623 (134) |
| Education-related activities | Homework | 952 (28.95%) | 102 (83) |
|  | In class | 61 (1.86%) | 245 (208) |
|  | School breaks | 24 (0.73%) | 117 (233) |
|  | School clubs | 7 (0.21%) | 179 (204) |
|  | Detention | 0 (0%) | 0 (0) |
| Personal care, travel and house chores | Eating a meal | 2,861 (87.01%) | 68 (54) |
|  | Personal care (including taking a shower/bath, grooming, getting dressed etc.) | 2,647 (80.50%) | 66 (65) |
|  | Travel by car, van (including vehicles owned by friends and family) | 1,364 (41.48%) | 85 (83) |
|  | Cooking, cleaning, and shopping for the household | 951 (28.92%) | 65 (65) |
|  | Eating a snack or having a drink | 1,411 (42.91%) | 32 (29) |
|  | Eating or drinking in a restaurant or café | 498 (15.15%) | 70 (51) |
|  | Travel by bus, taxi, tube, plane | 307 (9.34%) | 94 (109) |
|  | Pet care | 430 (13.08%) | 55 (62) |
|  | Paid work (including paid babysitting and paid work for the family) | 113 (3.44%) | 191 (204) |
|  | Looking after brothers, sisters, other children in the household | 150 (4.56%) | 89 (90) |
|  | Fixing things around the house, fixing bike, gardening | 158 (4.81%) | 81 (102) |
|  | Unpaid work for family or other non-household members (e.g. help in family business) | 76 (2.31%) | 147 (137) |
|  | Looking after parent or other adult in the household (medical or personal care) | 16 (0.49%) | 75 (80) |
| Physical activities | Other physical exercise (e.g. dancing, keeping fit) and other sports (e.g. skateboarding, gymnastics) | 560 (17.03%) | 108 (126) |
|  | Team ball games and training (e.g. football, hockey) | 344 (10.46%) | 151 (122) |
|  | Jogging, running, walking, hiking | 574 (17.46%) | 86 (92) |
|  | Travel by physically active means (walk, bike etc.) | 564 (17.15%) | 57 (62) |
|  | Swimming and other water sports | 127 (3.86%) | 143 (104) |
|  | Individual ball games and training (e.g. tennis, badminton) | 163 (4.96%) | 94 (78) |
|  | Cycling | 133 (4.05%) | 98 (91) |
| Social and hobbies | Speaking, socialising face-to-face | 1,627 (49.48%) | 146 (130) |
|  | Other activities not listed | 1,062 (32.30%) | 143 (144) |
|  | Did nothing, just relaxing, bored, waiting | 919 (27.95%) | 93 (105) |
|  | Shopping (including window shopping, hanging out at shopping centre) | 484 (14.72%) | 122 (98) |
|  | Hobbies, arts and crafts, musical activities, writing stories, poetry etc. | 529 (16.09%) | 108 (97) |
|  | Reading (not for school) | 449 (13.66%) | 86 (78) |
|  | Cinema, theatre, performance, gig etc. | 141 (4.29%) | 183 (104) |
|  | Religious activities (including going to places of worship, praying etc.) | 201 (6.11%) | 119 (85) |
|  | Attending live sporting events | 71 (2.16%) | 177 (136) |
|  | Volunteering | 40 (1.22%) | 168 (148) |
|  | Exhibition, museum, library, other cultural events | 45 (1.37%) | 133 (157) |

*Notes:* ^a^ Participation rate: cohort members spending >0 minutes on each activity; ^b^ Daily minutes spent on each activity by those participating.

**Table S2: Summary statistics of SDQ scores, split by gender**

|  | **All (N=3,288)** | **Males (n=1,474)** | **Females (n=1,814)** |
| --- | --- | --- | --- |
| **Age 14** |  |  |  |
| Conduct score ^a^ | 1.06 (1.34) | 1.07 (1.40) | 1.04 (1.28) |
| *Missing ^b^* | *62 (1.89%)* | *23 (1.56%)* | *39 (2.15%)* |
| Emotion score | 1.74 (1.98) | 1.40 (1.78) | 2.03 (2.09) |
| *Missing* | *62 (1.89%)* | *24 (1.63%)* | *38 (2.09%)* |
| Hyperactivity score | 2.40 (2.15) | 2.84 (2.28) | 2.05 (1.97) |
| *Missing* | *64 (1.95%)* | *24 (1.63%)* | *40 (2.21%)* |
| Peer score | 1.51 (1.69) | 1.61 (1.76) | 1.42 (1.62) |
| *Missing* | *61 (1.86%)* | *23 (1.56%)* | *38 (2.09%)* |
| Total difficulties score | 6.70 (5.18) | 6.92 (5.28) | 6.53 (5.09) |
| *Missing* | *65 (1.98%)* | *24 (1.63%)* | *41 (2.26%)* |
| Prosocial score | 8.60 (1.65) | 8.36 (1.73) | 8.78 (1.55) |
| *Missing* | *62 (1.89%)* | *24 (1.63%)* | *38 (2.09%)* |
|  |  |  |  |
| **Age 17** |  |  |  |
| Conduct score | 0.90 (1.28) | 0.92 (1.31) | 0.89 (1.25) |
| *Missing* | *573 (17.43%)* | *246 (16.69%)* | *327 (18.03%)* |
| Emotion score | 1.78 (2.08) | 1.33 (1.79) | 2.16 (2.23) |
| *Missing* | *574 (17.46%)* | *246 (16.69%)* | *328 (18.08%)* |
| Hyperactivity score | 1.99 (2.01) | 2.24 (2.08) | 1.78 (1.92) |
| *Missing* | *576 (17.52%)* | *248 (16.82%)* | *328 (18.08%)* |
| Peer score | 1.56 (1.67) | 1.64 (1.70) | 1.49 (1.65) |
| *Missing* | *574 (17.46%)* | *246 (16.69%)* | *328 (18.08%)* |
| Total difficulties score | 6.23 (5.21) | 6.12 (5.12) | 6.32 (5.28) |
| *Missing* | *576 (17.52%)* | *248 (16.82%)* | *328 (18.08%)* |
| Prosocial score | 8.64 (1.66) | 8.41 (1.77) | 8.83 (1.54) |
| *Missing* | *573 (17.43%)* | *246 (16.69%)* | *327 (18.03%)* |

*Notes*: ^a^ Mean and standard deviation for each variable. ^b^ Number and proportion (%) of cohort members.

**Table S3: Summary statistics of demographic, socioeconomic and health characteristics, split by gender**

|  | **All (N=3,288)** | **Males (n=1,474)** | **Females (n=1,814)** |
| --- | --- | --- | --- |
| **Age** | 13.76 (0.45) | 13.75 (0.46) | 13.77 (0.44) |
| *Missing* | *0 (0.00%)* | *0 (0.00%)* | *0 (0.00%)* |
| **Ethnicity** |  |  |  |
| White | 2,802 (85.22%) | 1,243 (84.33%) | 1,559 (85.94%) |
| Mixed | 125 (3.80%) | 68 (4.61%) | 57 (3.14%) |
| Indian | 90 (2.74%) | 48 (3.26%) | 42 (2.32%) |
| Pakistani and Bangladeshi | 140 (4.26%) | 54 (3.66%) | 86 (4.74%) |
| Black or Black British | 58 (1.76%) | 33 (2.24%) | 25 (1.38%) |
| Other Ethnic group | 56 (1.70%) | 19 (1.29%) | 37 (2.04%) |
| *Missing* | *17 (0.52%)* | *9 (0.61%)* | *8 (0.44%)* |
| **Obesity** |  |  |  |
| No | 3,012 (91.61%) | 1,384 (93.89%) | 1,628 (89.75%) |
| Yes | 193 (5.87%) | 79 (5.36%) | 114 (6.28%) |
| *Missing* | *83 (2.52%)* | *11 (0.75%)* | *72 (3.97%)* |
| **Family income** | | | |
| Lower quintile | 286 (8.70%) | 109 (7.39%) | 177 (9.76%) |
| Second quintile | 400 (12.17%) | 163 (11.06%) | 237 (13.07%) |
| Third quintile | 662 (20.13%) | 290 (19.67%) | 372 (20.51%) |
| Fourth quintile | 905 (27.52%) | 423 (28.70%) | 482 (26.57%) |
| Highest quintile | 1,032 (31.39%) | 487 (33.04%) | 545 (30.04%) |
| *Missing* | *3 (0.09%)* | *2 (0.14%)* | *1 (0.06%)* |
| **Parental education levels** | | | |
| No degree | 131 (3.98%) | 59 (4.00%) | 72 (3.97%) |
| NVQ level 1 -- low | 95 (2.89%) | 39 (2.65%) | 56 (3.09%) |
| NVQ level 2 | 537 (16.33%) | 231 (15.67%) | 306 (16.87%) |
| NVQ level 3 | 417 (12.68%) | 174 (11.80%) | 243 (13.40%) |
| NVQ level 4 | 1,396 (42.46%) | 623 (42.27%) | 773 (42.61%) |
| NVQ level 5 -- high | 706 (21.47%) | 345 (23.41%) | 361 (19.90%) |
| *Missing* | *6 (0.18%)* | *3 (0.20%)* | *3 (0.17%)* |
| **Parental employment status** | | | |
| Neither parent working | 234 (7.12%) | 91 (6.17%) | 143 (7.88%) |
| Only one parent working | 976 (29.68%) | 432 (29.31%) | 544 (29.99%) |
| Both parents working | 2,078 (63.20%) | 951 (64.52%) | 1,127 (62.13%) |
| *Missing* | *0 (0.00%)* | *0 (0.00%)* | *0 (0.00%)* |
| **Home ownership** | | | |
| No | 708 (21.53%) | 300 (20.35%) | 408 (22.49%) |
| Yes | 2,546 (77.43%) | 1,159 (78.63%) | 1,387 (76.46%) |
| *Missing* | *34 (1.03%)* | *15 (1.02%)* | *19 (1.05%)* |
| **Two-parent household** | | | |
| No | 607 (18.46%) | 254 (17.23%) | 353 (19.46%) |
| Yes | 2,681 (81.54%) | 1,220 (82.77%) | 1,461 (80.54%) |
| *Missing* | *0 (0.00%)* | *0 (0.00%)* | *0 (0.00%)* |
| **Index of Multiple Deprivation** | 5.97 (2.90) | 6.16 (2.86) | 5.81 (2.92) |
| *Missing* | *6 (0.18%)* | *4 (0.27%)* | *2 (0.11%)* |

*Notes*: Mean and standard deviation for continuous variables, including age and IMD. Number and proportion (%) of cohort members in each category for binary and categorical variables, including ethnicity, obesity, family income, parental education levels, parental employment status, home ownership and two-parent households.

**Table S4: Model fit statistics for the optimal number of profiles in LPA**

| **Model types** | **AIC** | **BIC** | **ssaBIC** | **Entropy** |
| --- | --- | --- | --- | --- |
| 2-profile | 158296.2 | 158412.1 | 158386.13 | 0.73154253 |
| 3- profile | 157205.2 | 157363.8 | 157345.11 | 0.78891309 |
| 4- profile | 156277.4 | 156478.7 | 156467.31 | 0.79077607 |
| 5- profile | 156367.5 | 156611.4 | 156607.36 | 0.89482866 |

*Notes*: AIC: Akaike's Information Criterion; BIC: Bayesian Information Criterion; ssaBIC: Sample Size Adjusted Bayesian Information Criterion.

**Table S5: Average minutes per day spent on each activity domain by latent profiles**

| **Activity domains** | **‘Frequent digital users’**  **(n=691)** | **‘Physically active, moderate digital users’**  **(n=1,962)** | **‘In-person socialisers, low digital users’**  **(n=635)** |
| --- | --- | --- | --- |
| Sleep | 589 (126) | 649 (138) | 569 (139) |
| Digital time | 567 (124) | 215 (116) | 125 (113) |
| Personal care, travel and household chores | 150 (91) | 262 (153) | 191 (124) |
| Social and hobbies | 81 (90) | 168 (109) | 504 (128) |
| Physical activities | 36 (64) | 97 (129) | 39 (68) |
| Education-related activities | 17 (45) | 50 (101) | 12 (33) |

*Notes*: Results shown as average time spent (measured as minutes per day) and standard deviations for those who participated in activities.

**Table S6: Summary statistics of SDQ scores, demographic, socioeconomic and health characteristics by latent profiles**

|  | **‘Frequent digital users’**  **(n=691)** | **‘Physically active, moderate digital users’**  **(n=1,962)** | **‘In-person socialisers, low digital users’**  **(n=635)** |
| --- | --- | --- | --- |
| **Demographic and socioeconomic characteristics** | | | |
| Age | 13.77 (0.45) | 13.76 (0.45) | 13.74 (0.45) |
| Proportion of females | 0.38 (0.48) | 0.58 (0.49) | 0.66 (0.47) |
| Proportion of white ethnicity | 0.86 (0.34) | 0.85 (0.36) | 0.88 (0.32) |
| Proportion of obesity | 0.07 (0.25) | 0.06 (0.23) | 0.06 (0.24) |
| Proportion of two-parent households | 0.78 (0.41) | 0.83 (0.38) | 0.82 (0.39) |
| Proportion of both parents employed | 0.56 (0.50) | 0.65 (0.48) | 0.65 (0.48) |
| Proportion of high household income | 0.56 (0.50) | 0.61 (0.49) | 0.57 (0.49) |
| Proportion of high parental education | 0.73 (0.45) | 0.78 (0.41) | 0.77 (0.42) |
| Proportion of home ownership | 0.74 (0.44) | 0.80 (0.40) | 0.77 (0.42) |
| IMD deciles | 5.63 (2.91) | 6.09 (2.88) | 5.97 (2.91) |
|  |  |  |  |
| **SDQ scores** |  |  |  |
| Total score |  |  |  |
| *Age 14* | 7.47 (5.54) | 6.44 (4.98) | 6.69 (5.31) |
| *Age 17* | 7.06 (5.73) | 5.82 (4.86) | 6.55 (5.50) |
| Conduct score |  |  |  |
| *Age 14* | 1.15 (1.41) | 1.01 (1.28) | 1.09 (1.43) |
| *Age 17* | 1.00 (1.36) | 0.85 (1.23) | 0.96 (1.35) |
| Emotion score |  |  |  |
| *Age 14* | 1.88 (2.10) | 1.69 (1.92) | 1.76 (2.02) |
| *Age 17* | 1.87 (1.87) | 1.45 (1.58) | 1.53 (1.66) |
| Hyperactivity score |  |  |  |
| *Age 14* | 2.61 (2.20) | 2.32 (2.13) | 2.44 (2.15) |
| *Age 17* | 2.24 (2.17) | 1.87 (1.93) | 2.07 (2.04) |
| Peer score |  |  |  |
| *Age 14* | 1.83 (1.80) | 1.43 (1.64) | 1.40 (1.66) |
| *Age 17* | 1.87 (1.87) | 1.45 (1.58) | 1.53 (1.66) |
| Prosocial score |  |  |  |
| *Age 14* | 8.36 (1.73) | 8.68 (1.60) | 8.58 (1.67) |
| *Age 17* | 8.39 (1.74) | 8.73 (1.62) | 8.60 (1.67) |

*Notes:* Results shown as mean and standard deviations in parentheses.

**Table S7: Associations between latent profiles and SDQ total and subscale scores using multiple imputation**

|  | **Unadjusted** | | **Partially adjusted ^a^** | **Fully adjusted ^b^** | |  |
| --- | --- | --- | --- | --- | --- | --- |
| **SDQ total score** |  |  | | |  | |
| Frequent digital users | 1.23 | 0.53 | | | 0.60 | |
| 95% confidence interval | [0.75,1.72] | [0.16,0.90] | | | [0.22,0.97] | |
| *p value* | *0.000* | *0.006* | | | *0.002* | |
| In-person socialisers | 0.81 | 0.65 | | | 0.61 | |
| 95% confidence interval | [0.30,1.32] | [0.25,1.05] | | | [0.20,1.01] | |
| *p value* | *0.002* | *0.002* | | | *0.003* | |
| **SDQ conduct score** |  |  | | |  | |
| Frequent digital users | 0.13 | 0.06 | | | 0.05 | |
| 95% confidence interval | [0.01,0.26] | [-0.05,0.16] | | | [-0.06,0.15] | |
| *p value* | *0.032* | *0.282* | | | *0.373* | |
| In-person socialisers | 0.12 | 0.08 | | | 0.08 | |
| 95% confidence interval | [-0.00,0.25] | [-0.03,0.19] | | | [-0.03,0.19] | |
| *p value* | *0.053* | *0.154* | | | *0.160* | |
| **SDQ emotional score** |  |  | | |  | |
| Frequent digital users | 0.30 | 0.19 | | | 0.26 | |
| 95% confidence interval | [0.11,0.49] | [0.03,0.35] | | | [0.10,0.42] | |
| *p value* | *0.002* | *0.018* | | | *0.002* | |
| In-person socialisers | 0.37 | 0.33 | | | 0.27 | |
| 95% confidence interval | [0.16,0.57] | [0.14,0.51] | | | [0.09,0.46] | |
| *p value* | *0.001* | *0.001* | | | *0.004* | |
| **SDQ hyperactivity score** |  |  | | |  | |
| Frequent digital users | 0.39 | 0.20 | | | 0.19 | |
| 95% confidence interval | [0.20,0.57] | [0.06,0.35] | | | [0.04,0.35] | |
| *p value* | *0.000* | *0.007* | | | *0.013* | |
| In-person socialisers | 0.22 | 0.15 | | | 0.15 | |
| 95% confidence interval | [0.02,0.41] | [-0.01,0.30] | | | [-0.01,0.30] | |
| *p value* | *0.027* | *0.065* | | | *0.064* | |
| **SDQ peer score** |  |  | | |  | |
| Frequent digital users | 0.41 | 0.19 | | | 0.18 | |
| 95% confidence interval | [0.25,0.57] | [0.05,0.33] | | | [0.03,0.32] | |
| *p value* | *0.000* | *0.008* | | | *0.016* | |
| In-person socialisers | 0.10 | 0.12 | | | 0.12 | |
| 95% confidence interval | [-0.05,0.26] | [-0.01,0.26] | | | [-0.01,0.26] | |
| *p value* | *0.191* | *0.072* | | | *0.069* | |
| **SDQ prosocial score** |  |  | | |  | |
| Frequent digital users | -0.33 | -0.16 | | | -0.12 | |
| 95% confidence interval | [-0.49,-0.17] | [-0.29,-0.02] | | | [-0.26,0.02] | |
| *p value* | *0.000* | *0.025* | | | *0.084* | |
| In-person socialisers | -0.14 | -0.09 | | | -0.10 | |
| 95% confidence interval | [-0.30,0.02] | [-0.22,0.05] | | | [-0.24,0.04] | |
| *p value* | *0.076* | *0.195* | | | *0.147* | |
| **Number of observations** | **3288** | | **3288** | **3288** | |  |

*Notes:* ^a^ Adjusted for baseline SDQ scores; ^b^ Adjusted for baseline SDQ scores, and demographic and socio-economic status. ^c^ The reference group in latent profiles was physically active, moderate digital users.

Results shown as marginal effects β and 95% confidence intervals in parentheses, along with p values.
